# Information-Theoretic Trigger Surprisal and Future Headache Activity

**DOI:** 10.1001/jamanetworkopen.2025.42944

**Published:** 2025-11-11

**Authors:** Dana P. Turner, Twinkle Patel, Emily Caplis, Timothy T. Houle

**Affiliations:** 1 Department of Anesthesia, Critical Care and Pain Medicine, Massachusetts General Hospital, Harvard Medical School, Boston; 2 Department of Neurology, UMass, Worcester, Massachusetts

## Abstract

**Importance:**

Identifying migraine triggers is a common goal for individuals with migraine but remains challenging due to the vast number of possible triggers and their fluctuating nature. A measurement system quantified through information-theoretic surprisal (ie, the negative logarithm of the probability of an observed exposure) could be valuable for evaluating the wide range of possible triggers for future migraine attacks.

**Objective:**

To evaluate the association between surprisal and future headache attacks.

**Design, Setting, and Participants:**

This cohort study conducted from April 2021 to December 2024 included 109 participants with migraine who completed diary entries of potential migraine triggers and migraine attacks within 12 and 24 hours after trigger exposure twice daily for up to 28 days.

**Exposure:**

Diary items captured exposure to potential behavioral, emotional, and environmental migraine triggers. To quantify the unexpectedness of a participant’s daily experiences, total surprisal scores measured in bits of information was calculated using within-person empirical probability distributions to reflect how atypical each day’s experiences were.

**Main Outcomes and Measures:**

The primary outcomes were occurrence of a headache attack within 12 and 24 hours (0, no attack; 1, attack) after trigger exposure. A headache attack was defined as any self-reported headache with pain greater than 0 on a 0 to 10 numeric rating scale where 0 was no pain and 10 was the worst pain, and any pattern of secondary symptoms (eg, photophobia and phonophobia). Intraclass correlation coefficients and variance components were calculated to quantify between-person differences in baseline risk and the strength of the surprisal association.

**Results:**

A total of 109 participants were included in the analysis. Participants’ median age was 35 (26.0-46.0) years, and the majority were female (102 [93.5%]). One hundred and four participants had complete diaries (5176 diary entries) and experienced headaches on 1518 of 5145 days (29.5%). Higher surprisal was significantly associated with increased migraine risk within 12 hours (odds ratio [OR], 1.86; 95% CI, 1.12-3.08; *P* = .02) and 24 hours (OR, 2.15; 95% CI, 1.44-3.20; *P* < .001), with a higher OR indicating a stronger association observed at the longer interval. The association between current surprisal and migraine onset was moderated by recent surprisal history and exhibited nonlinear properties at 12 hours. Random-effects models revealed substantial between-person variability and, after adjustment, the association between total surprisal and headache attack risk was attenuated (OR, 1.56; 95% CI, 1.01-2.40; *P* = .04 for 12 hours and OR, 1.88; 95% CI, 1.27-2.79; *P* = .002 for 24 hours).

**Conclusions and Relevance:**

In this cohort study, surprisal offered a novel, individualized measure of trigger unpredictability that was associated with short-term migraine risk. Incorporating surprisal into digital tools may improve personalized prevention strategies, moving beyond static trigger lists to a dynamic, context-aware model of migraine self-management.

## Key Points

**Question:**

Is there an association between information-theoretic trigger surprisal and future headache activity?

**Findings:**

In this cohort study including 109 participants, total surprisal score was associated with future migraine risk at 12 hours and 24 hours.

**Meaning:**

The findings of this study underscore the value of a person-centered, information-theoretic approach to understanding migraine triggers, one that moves beyond static lists of potential causes to account for the unpredictable and context-sensitive nature of daily life.

## Teaser

This cohort study assesses the association between information-theoretic trigger surprisal and future headache activity in patients with migraine.

## Introduction

To mitigate the burden of migraine, individuals often strive to identify triggers of their attacks.^1,2,3^ In practice, this process relies on biopsychosocial covariation assessment, in which fluctuations of a suspected trigger are examined with variations in the pattern of migraine occurrence.^4,5,6,7,8,9^ However, this task is difficult, particularly when the number of potential triggers is vast.^10,11^ Hundreds of factors have been proposed as migraine triggers, ranging from dietary and environmental factors to physiological and psychological stressors, making it nearly impossible for individuals to isolate any single cause with certainty.^2,3,6^

While well-controlled experimental designs could help clarify these associations, they are difficult to implement in everyday life. Instead, people often rely on natural experiments in which both potential causes and migraine outcomes vary freely, making it challenging to determine causal associations .^8,11,12^ Without a systematic approach, this reliance on observational assessment may lead to inaccurate conclusions that may not be scientifically valid.^13^

A robust measurement system capable of evaluating the wide range of possible triggers could provide substantial value.^7^ Such a system would need to accurately quantify the variability in an individual’s exposure to different migraine triggers over time and demonstrate a reliable association between these exposures and the likelihood of near-future migraine attacks. Such a comprehensive measurement framework could improve identification of factors associated with migraine risk and empower individuals with more effective strategies for prevention and management.

We have proposed that headache triggers could be understood not only through their unique physiological mechanisms but also by the degree of surprise they present to individuals.^14^ Using information theory, this work demonstrated that rare or unexpected values of common triggers, such as caffeine and alcohol consumption, stress, and mood disturbances, were consistently associated with increased headache activity.^15,16^ Summing these individual surprisals into a total trigger surprise score provided stronger discrimination between headache and nonheadache days than any trigger alone.^14^ More recent research has focused on developing a comprehensive migraine trigger measurement system using surprisal and entropy measures, revealing substantial heterogeneity in exposure patterns within individuals.^17^ A small number of principal components explained most of this variability, suggesting that migraine trigger exposure may be effectively characterized along a few key dimensions. Together, these findings highlight surprisal as a valuable metric for quantifying the unpredictability of trigger exposures and improving headache forecasting.

The present study aims to further examine the association between trigger surprisal and future migraine attacks. We hypothesized that the original association observed between surprisal and headache activity^14^ could be replicated in prospectively collected data.^17^ We also describe the application of this migraine trigger measurement system by examining the associations of fluctuations in surprisal measurements and near-future migraine activity.

## Methods

This cohort study was approved by the institutional review board of Mass General Brigham. This study is the second preplanned primary analysis of these data. The study was conducted from April 2021 to December 2024 and is reported according to the Strengthening the Reporting of Observational Studies in Epidemiology (STROBE) reporting guideline.^18^ Participants were recruited through an institutional online recruitment platform, public transportation locations, and flyers. Interested individuals underwent telephone screening to determine eligibility. The inclusion criteria were having an *International Classification of Headache Disorders, 3rd Edition*^19^ diagnosis of migraine with or without aura, experiencing 4 to 14 headache days per month, and being 18 to 65 years of age. Exclusion criteria included secondary headache disorder, chronic daily or medication overuse headache, recent change in headache symptoms, and pregnancy.

Eligible participants were invited for an in-person or virtual enrollment session. Before participation, individuals completed an electronic informed consent process in Research Electronic Data Capture (REDCap; Vanderbilt University).^20^ They completed enrollment questionnaires in REDCap (eg, demographic and headache information, Migraine Disability Assessment [score range: 0-270, with higher scores indicating greater disability).^21^ Demographic information included age, sex, and self-reported race and ethnicity, which included the following categories: American Indian or Alaska Native, Asian, Black, Hispanic, and White. Data on race and ethnicity were included in the analysis as part of standard National Institutes of Health reporting.

Participants received instructions for at-home procedures, which involved twice-daily diary entries, 1 morning and 1 evening, for 28 days. The diaries, completed in REDCap, required approximately 5 to 10 minutes. On completion of study procedures, participants took part in a final session and completed another series of questionnaires similar to those in the enrollment session. Additional questionnaires were completed at study enrollment and completion but are not reported here.

### Daily Diary Items

The electronic diaries captured exposure to a wide range of potential migraine triggers, headache activity, and medication use. The individual items and their variability were described previously.^17^ Given the nature of different trigger constructs, distinct sets of triggers were assessed in the morning (AM) and evening (PM). The AM diary focused on sleep-related behaviors, including duration, quality, awakenings, bedtime, and wake time. It also included questions about late-night meals, weather influences, and mood state assessed with the Profile of Mood States Short Form, which measures 6 mood states (tension-anxiety, depression-dejection, anger-hostility, vigor-activity, fatigue-inertia, and confusion-bewilderment) using a Likert-type scale.^22^ The PM diary similarly assessed mood state with the Profile of Mood States Short Form but also captured data on commonly reported food and drink triggers, environmental exposures, meal patterns or missed meals, and weather influences. Additionally, the PM diary included measures of daily stressors from the Daily Stress Inventory, which measures exposure to 58 common daily hassles using a Likert-type scale.^23^

### Migraine Trigger Risk Scoring System

To quantify the unexpectedness of an individual’s daily response pattern, we applied the concept of surprisal from information theory.^14,16^ Surprisal is defined as the negative logarithm of the probability of an observed exposure (−log_2_[p_exposure_]) and reflects how unlikely that outcome is under a specified probability distribution. Empirical probability distributions were constructed for each individual using their observed responses. For each diary period, the surprisal scores (bits) of the individual diary items were computed based on these person-specific distributions. The total surprisal score was then calculated as the sum of item-level surprisal scores for that day and scaled by the number of items to yield a mean surprisal score per item, expressed in bits of information. This scaling allows for missing items to be ignored and for AM diaries to be scaled similar to PM diaries, despite the latter having more items as described by Turner DP et al.^17^ This approach provides a within-person measure of how atypical a given day’s responses are relative to that individual’s behavioral patterns.

In our previous work,^17^ the calculation of total surprisal incorporated 2 components: surprisal based on the magnitude of each measurement and surprisal associated with the change from the preceding value. This required estimating and summing surprisal values from 2 within-person empirical distributions: the distribution of observed values and the distribution of within-person changes (ie, X_t_ − X_t −1_, where X_t_ represents the trigger exposure today and X_t-1_ represents the trigger exposure at the previous diary entry) for each variable. In the current analysis, we simplify this approach by omitting the change-based component of surprisal. Instead, we include a lagged total surprisal term (Surprisal_t −1_) in the statistical models to account for short-term fluctuations in surprisal over time.

### Outcomes

The primary outcomes for this analysis are 2 binary variables indicating the occurrence of a headache attack within a specified future time window. Specifically, we define a dichotomous outcome (0, no attack; 1, attack) representing whether a headache attack occurs within the subsequent 12 and 24 hours. An attack was defined as any self-reported headache with pain greater than 0 on the numeric rating scale where 0 is no pain and 10 is the worst pain, and any pattern of secondary symptoms (eg, photophobia, phonophobia). These outcomes were aligned to each diary entry time point (eg, 12 hours from the AM entry) and allowed us to evaluate short-term prospective associations between daily surprisal and the onset of headache attacks over 2 clinically relevant temporal horizons.

### Statistical Analyses

The originally planned sample size of 200 provided a 95% CI precision of 4.16% for an observed proportion of 10%. However, the COVID-19 pandemic limited participant enrollment to 109, resulting in a broader estimated 95% CI precision of 5.70%, which was deemed sufficient for evaluating the study objectives. All statistical analyses were conducted using R, version 4.4.1 (R Project for Statistical Computing) and RStudio (Posit PBC). Mean (SD) or median (IQR) was used to describe continuous variables, while frequencies (%) were used for categorical variables. To examine the association between daily surprisal scores and the likelihood of subsequent migraine attacks, generalized linear mixed-effects models were used. These models accounted for repeated diary entries within individuals by including a random intercept for each participant. In the primary analyses, the fixed effects included the total surprisal score at AM or PM, with random slopes for surprisal to capture interindividual variability in the surprisal-headache association. Models were estimated separately for the 2 binary outcomes using a binomial likelihood and logit link. Temporal dependencies were modeled by including the lagged headache outcome from the previous diary entry. We also tested for nonlinear and interaction effects by including a squared term for current surprisal and its interaction with the lagged surprisal score. Model assumptions were verified through residual diagnostics. There were 710 of 5886 (12.1%) missing diary entries, resulting in an adherence rate of 88%. Missing data were ignored for analysis, with all models conducted on complete cases. A 2-tailed *P* < .05 was considered statistically significant for all hypothesis tests. Intraclass correlation coefficients and variance components were calculated to quantify between-person differences in baseline risk and the strength of the surprisal association. In sensitivity analyses, models were adjusted for each participant’s mean surprisal score to better isolate within-person effects.

## Results

The study enrolled 109 participants with a median (IQR) age of 35 (26-46) years; 102 were female (93.5%) and 7 were male (6.5%). Participants self-reported race as follows: 3 were American Indian or Alaska Native (2.8%), 9 were Asian (8.3%), 6 were Black (5.5%), and 91 were White (83.5%) individuals; 11 individuals (10.2%) reported Hispanic ethnicity. Fifty-nine participants (54.6%) were single, 44 (40.7%) were married, 2 (1.9) were widowed, and 3 (2.8) were divorced or separated. Of the 109 individuals enrolled, 104 completed twice-daily electronic entries for approximately 28 days, yielding 5176 total diaries. All participants had migraine, with a median (IQR) headache frequency of 8 (5-12) days per month and a median intensity of 7 of 10 (6-8), with intensity measured using a numeric rating scale of 0 to 10, with higher scores representing higher intensity. The median (IQR) Migraine Disability Assessment score of 24 (13-36) indicated moderate to severe disability.

### Distribution of Surprisal Scores

Table 1 presents the pooled distribution of surprisal scores for all participant days. Higher surprisal scores were observed in the presence of a current headache (range, 0.06-0.10 bits) and when a future headache would develop (range, 0.01-0.06 bits). The highest mean surprisal scores were identified from the PM diary entries. These differences indicate that when pooled across individuals, the surprisal scores allowed modest discrimination across headache days.

**Table 1.** Daily Characteristics of Scaled Surprisal Scores^a^

Current headache	Future headache at 12 h		Future headache at 24 h	
	No	Yes	No	Yes
**AM diary**				
No	0.53 (0.18)	0.56 (0.19)	0.53 (0.18)	0.55 (0.18)
Yes	0.61 (0.21)	0.62 (0.19)	0.61 (0.22)	0.62 (0.18)
**PM diary**				
No	0.62 (0.24)	0.64 (0.24)	0.60 (0.23)	0.65 (0.25)
Yes	0.70 (0.26)	0.74 (0.24)	0.67 (0.25)	0.73 (0.25)

^a^ Values presented are mean (SD) surprisal scores (bits of information).

### Association of Total Surprisal Score and Future Headache Activity

**Figure 1.**
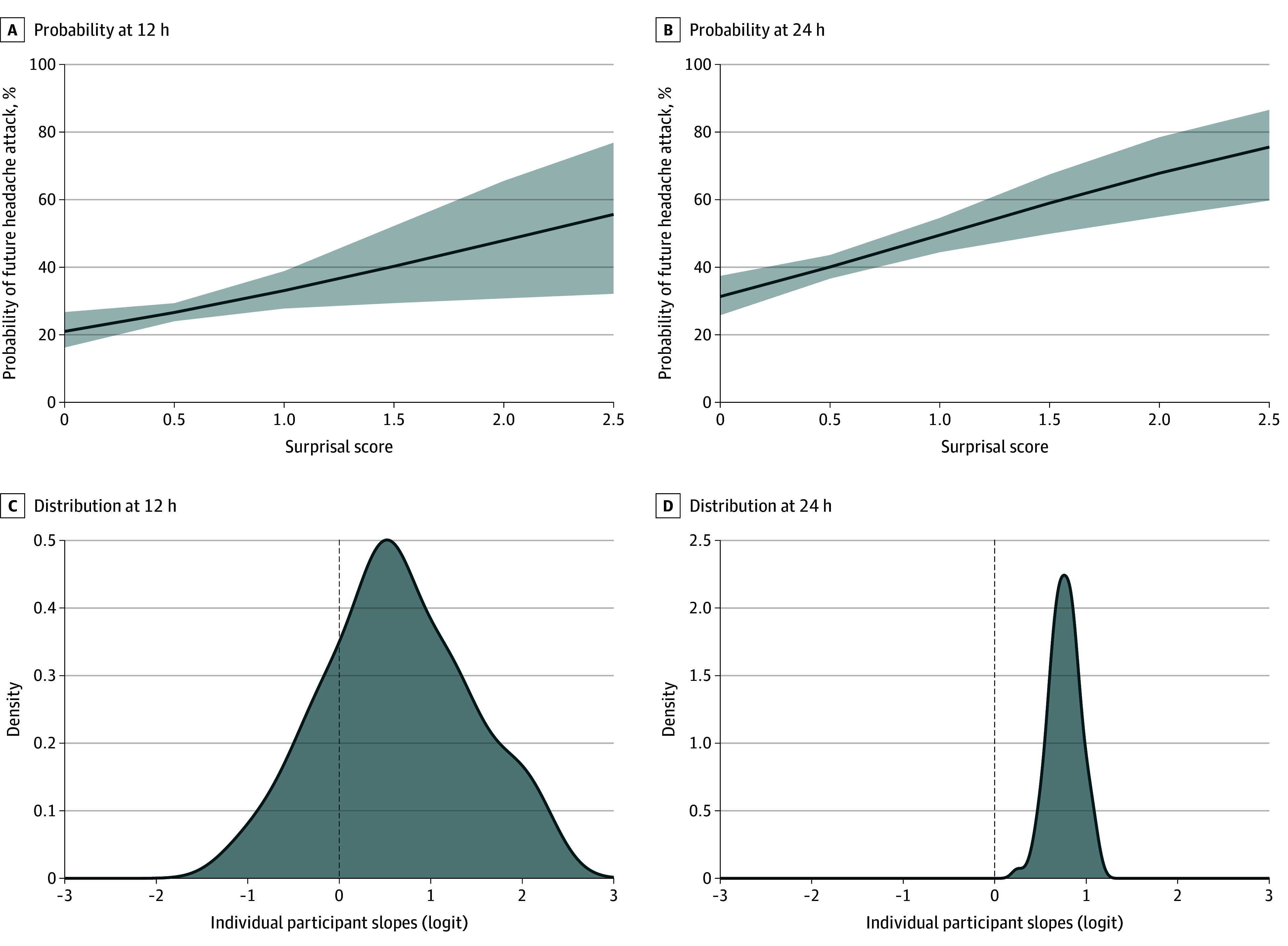
Probability of Future Headache Attack as a Function of Current Surprisal Score A and B, Association between current surprisal score and the probability of near-future headache attack. C and D, The distributions of individual surprisal slopes from the mixed-effects models on the logit scale highlight variability in person-level associations. The dashed lines denote zero slope. Shaded areas represent 95% CIs.

Participants experienced a headache on 1518 of 5145 days (29.5%) with complete diary information. The total surprisal score was associated with both 12-hour and 24-hour headache attack risk, with odds ratios (ORs) of 1.86 (95% CI, 1.12-3.08; *P* = .02) and 2.15 (95% CI, 1.44-3.20; *P* < .001), respectively. This means that for each bit increase in surprisal, the odds of a future headache increased by 86% and 115%, with increasing odds at 24 hours compared with 12 hours. The random variance components indicate substantial between-person variability in how surprisal influences headache risk. Specifically, the random slope variance (τ_11_) for surprisal was 2.00 at 12 hours but was 0.31 for 24 hours (Table 2 and Figure 1). The negative correlation between random intercept and surprisal slope (ρ_01_ = −0.81 for 12 hours; ρ_01_ = −0.50 for 24 hours) further implies that individuals with higher baseline headache risk exhibited a weaker association between surprisal and headache attack likelihood. The intraclass correlation coefficient of 0.11 for both models indicated that 11% of the total variance was attributable to individual differences.

**Table 2.** Association of Surprisal Total Score and Future Headache Attack^a^

	Future headache at 12 h		Future headache at 24 h	
	OR (95% CI)	*P* value	OR (95% CI)	*P* value
Factors				
Intercept	0.68 (0.49-0.96)	.027	0.53 (0.39-0.71)	<.001
Current headache				
No	1 [Reference]	NA	1 [Reference]	NA
Yes	1.88 (1.61-2.20)	<.001	1.64 (1.43-1.89)	<.001
Diary type				
AM	1 [Reference]	NA	1 [Reference]	NA
PM	0.47 (0.41-0.55)	<.001	0.82 (0.72-0.93)	.002
Surprisal	1.86 (1.12-3.08)	.02	2.15 (1.44-3.20)	<.001
Random effect^b^				
σ^2^	3.29	NA	3.29	NA
τ_00_	0.81	NA	0.54	NA
τ_11_	2.00	NA	0.31	NA
ρ_01_	−0.81	NA	−0.50	NA
ICC	0.11	NA	0.11	NA
No.	103	NA	104	NA
Observations	4530	NA	4947	NA
Marginal *R*^2^/conditional *R*^2^	0.054/0.157	NA	0.024/0.136	NA

Abbreviations: ICC, intraclass correlation coefficient; NA, not applicable; OR, odds ratio.

^a^ Each model is conducted based on the available sample size, which may differ across models due to missing data in either the outcome period (12 vs 24 hours) or any lagged terms.

^b^ The random effect σ^2^ represents total variance; τ_00_, variance component of intercept; τ_11_, variance component of slope; ρ_01_, correlation between intercept and slope.

In the sensitivity model, each individual’s mean surprisal score was further adjusted to better consider between-person variation. In these models, the association between total surprisal and attack risk was somewhat attenuated for 12 hours (OR, 1.56; 95% CI, 1.01-2.40; *P* = .04) and 24 hours (OR, 1.88; 95% CI, 1.27-2.79; *P* = .002). Overall, these results highlight surprisal as a key risk factor for headache onset, with individual differences potentially playing a critical role in shaping this association over time.

### Nonlinear and Contextual Associations of Total Surprisal Score and Future Headache Activity

**Figure 2.**
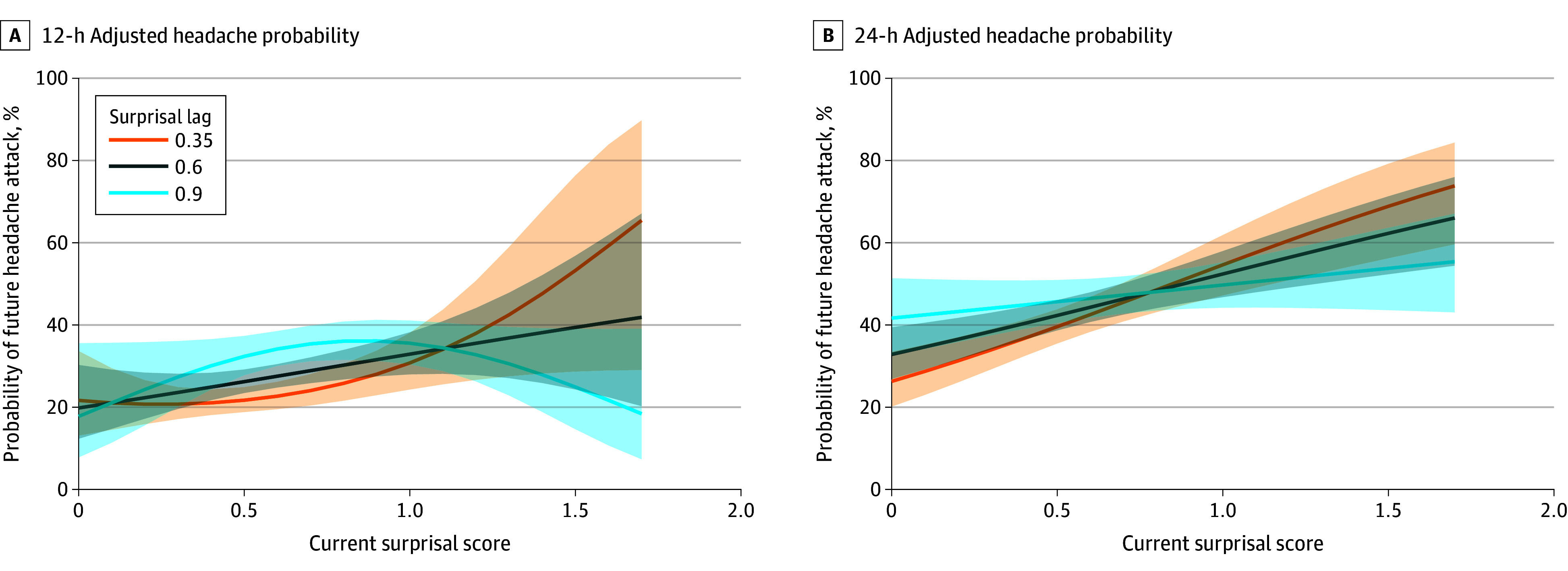
Probability of Future Headache Attack as a Function of Current Surprisal Based on Different Levels of Lagged Surprisal Score From the Previous Day The surprisal score from the previous day (lag) modulated the association between current surprisal score and headache risk. Shaded areas represent 95% CIs.

At 12 hours, there was a significant interaction between the current surprisal score and its lagged value, along with evidence supporting a nonlinear (quadratic) association. The best-fitting model included both linear and squared surprisal terms, the lagged surprisal term, and their interactions. Higher current surprisal was associated with an increased probability of a headache within 12 hours, but this probability varied depending on prior surprisal (Figure 2 and Table 3). When prior surprisal was low (lag = 0.35), the interaction of current surprisal was positive and nonlinear (ie, Surprisal × Surprisal_t-1_ interaction), with headache probability increasing steeply with higher surprisal. In contrast, at higher lag values (lag = 0.9), the association flattened or even reversed direction (ie, increasing surprisal became associated with decreased headache attack likelihood, Surprisal^2^ × Surprisal_t-1_ interaction). The interaction between the current surprisal squared and its lagged value was statistically significant (OR, 0.02; 95% CI, 0.00-0.43; *P* = .01), highlighting how recent context can modulate the association with momentary experiential dynamics.

**Table 3.** Contextual Associations of Surprisal Total Score and Future Headache Attack^a^

	Future headache at 12 h		Future headache at 24 h	
	OR (95% CI)	*P* value	OR (95% CI)	*P* value
Factors^b^				
Intercept	0.75 (0.25-2.31)	.62	0.28 (0.15-0.51)	<.001
Current headache (yes)				
No	1 [Reference]	NA	1 [Reference]	NA
Yes	1.86 (1.59-2.18)	<.001	1.63 (1.41-1.89)	<.001
Diary type				
AM	1 [Reference]	NA	1 [Reference]	NA
PM	0.51 (0.44-0.59)	<.001	0.80 (0.70-0.92)	.002
Surprisal	0.11 (0.00-2.84)	.18	5.95 (2.54-13.95)	<.001
Surprisal^2^	10.66 (1.12-101.15)	.04	NA	
Surprisal_t-1_	0.64 (0.09-4.37)	.65	3.54 (1.52-8.22)	.003
Surprisal × Surprisal_t-1_	137.48 (0.82-22 921.53)	.06	0.20 (0.07-0.59)	.004
Surprisal^2^ × Surprisal_t-1_	0.02 (0.00-0.43)	.01	NA	NA
Random effect^c^				
σ^2^	3.29	NA	3.29	NA
τ_00_	0.33	NA	0.43	NA
ICC	0.09	NA	0.12	NA
No. of participants	103	NA	103	NA
Observations	4440	NA	4440	NA
Marginal *R*^2^/conditional *R*^2^	0.080/0.164	NA	0.031/0.144	NA

Abbreviations: ICC, intraclass correlation coefficient; NA, not applicable; OR, odds ratio.

^a^ Each model is conducted based on the available sample size, which may differ across models due to missing data in either the outcome period (12 vs 24 hours) or any lagged terms.

^b^ The interaction terms (Surprisal × Suprisal_t-1_) indicate that recent experiences with surprisal alter the association of current experiences with surprisal, in either a linear or a nonlinear way. Surprisal is the current surprisal total score; surprisal^2^ is the square of the surprisal total score (surprisal x surprisal); surprisal_t-1_ is the surprisal total score from the previous diary entry.

^c^ The random effect σ^2^ represents total variance; τ_00_, variance component of intercept; τ_11_, variance component of slope; ρ_01_, correlation between intercept and slope.

In contrast, the 24-hour model demonstrated a simpler, largely linear pattern in the association between surprisal and headache probability. This model did not benefit from including a quadratic term, although it retained the lagged surprisal factor and its interaction with the current surprisal score (Surprisal × Surprisal_t-1_). Higher current surprisal was associated with a greater probability of headache 24 hours later, but the strength of this association decreased as the lagged surprisal increased (Figure 2 and Table 3). Specifically, at low lag values (lag = 0.35), surprisal had a steep positive slope, with increasing odds of headache attack (OR, 5.95; 95% CI, 2.54-13.95; *P* < .001). However, at higher lag values, this association was attenuated, consistent with a negative interaction between current surprisal and its lagged counterpart (OR, 0.20; 95% CI, 0.07-0.59; *P* = .004).

## Discussion

This cohort study evaluated the association between daily surprisal scores by quantifying the unexpectedness of daily experiences and the subsequent occurrence of migraine attacks within 12- and 24-hour periods. We found higher surprisal scores were associated with an increased risk of future headache attacks. These results support our initial hypothesis and replicate earlier findings^14^ in a new, prospectively collected dataset, demonstrating use of surprisal as a valuable metric for forecasting migraine risk.

The results of the present study provide evidence that the degree to which an individual’s experiences deviate from their usual patterns can be used to identify near-future migraine risk. For each unit increase in mean surprisal, the odds of a migraine attack rose, with a stronger association observed over the 24-hour period than the 12-hour period. This finding suggests that the association of daily experiences with migraine onset is not immediate in all cases and may accumulate or evolve. The association of current surprisal score with future headache risk was attenuated, or even reversed, when recent surprisal scores were already elevated, indicating a possible contextual adaptation or regulatory effect. These findings suggest that while current surprisal score is a robust risk factor for subsequent headache activity, its influence is temporally dynamic, moderated by recent history.

A key strength of this study lies in its examination of individual differences. The random slope models revealed considerable variability in how surprisal altered headache risk across participants. Some individuals appeared more sensitive to fluctuations in daily life, while others showed little to no association between surprisal and migraine. Interestingly, those with higher baseline headache risk tended to show weaker associations with surprisal. This pattern raises the possibility that migraine activity in these individuals may be affected by factors other than daily behavioral or environmental changes.

These findings suggest that surprisal may be a useful construct for advancing migraine self-management. Current trigger-tracking approaches often emphasize binary relationships between exposures and outcomes, potentially overlooking the dynamic and context-dependent nature of life experiences. By contrast, surprisal offers an integrative, continuous, person-centered measure that reflects how unusual a given day is for a specific individual. Embedding surprisal scores into digital headache diaries or wearable technologies could enable real-time risk forecasting and provide users with actionable insights.^9,24,25,26^ Novel interventions might focus not on avoiding specific triggers^27,28,29^ but on incorporating behavioral regularity or emotional regulation to control the experience of surprisal itself.

Our results extend prior work demonstrating the value of information-theoretic approaches in migraine research.^14,15,17^ Earlier studies showed that surprisal scores could discriminate headache days from nonheadache days and that these scores captured meaningful within-person variation in trigger exposures.^14^ Additionally, this study adds several critical dimensions by showing that surprisal is associated with different aspects of future risk (ie, 12 vs 24 hours). Furthermore, the use of lagged surprisal values and different times of day (ie, AM and PM) highlights the importance of temporal context, a feature that has received little attention in prior migraine forecasting models.^9,24^

### Limitations

Study limitations should be acknowledged. The final sample size was smaller than initially planned due to COVID-19 pandemic-related disruptions and precluded the consideration of specific subpopulations (eg, age and headache type). Additionally, the current analysis focused solely on magnitude-based surprisal, omitting the change-based component used in earlier work. While lagged surprisal helped account for short-term fluctuations, future models may benefit from further consideration of integrating both components. We also did not include potential covariates such as acute medication use, which may interact with surprisal to alter migraine risk. Furthermore, the use of retrospective self-report of headache attacks could be unreliable for some attacks, although the current study used limited periods (ie, 12-hour retrospection) to reduce the reliance on memory. Future research should develop methods to estimate surprisal prospectively, consider the implications of attack features or subgroup membership for associations between surprisal and headache attack, and consider ways to make the scoring method less burdensome (eg, the time required to complete hundreds of diary items could be prohibitive for some individuals).

## Conclusions

In this cohort study of the association between surprisal and future headache attacks, findings supported use of a surprisal scoring system as a dynamic, individualized metric capable of estimating short-term migraine risk. This finding underscores the value of a person-centered, information-theoretic approach to understanding migraine triggers, moving beyond static lists of potential causes to account for the unpredictable and context-sensitive nature of daily life. Incorporating measurement of surprisal into migraine forecasting tools could provide individuals with a more effective, personalized strategy for managing headache risk.

## Notes

Supplement.
Data Sharing Statement
