# Supplementary material for: Information-Theoretic Trigger Surprisal and Future Headache Activity
Source: JAMA Netw Open. 2025 Nov 11;8(11):e2542944. doi: 10.1001/jamanetworkopen.2025.42944 (PMC12606372; doi:10.1001/jamanetworkopen.2025.42944)
Supplement: Supplement. — Data Sharing Statement [file jamanetwopen-e2542944-s001.pdf]

Data Sharing Statement

Turner. Information-Theoretic Trigger Surprisal and Future Headache Activity. *JAMA Netw Open*. Published November 11, 2025. doi:10.1001/jamanetworkopen.2025.42944

Data
Data available: No
